# A Multicentre, 4‐Year Mirror‐Image Study Comparing the Effectiveness of Long‐Acting Injectable Antipsychotics in the Treatment of Bipolar Disorder: Results From the LAICO Study

**DOI:** 10.1111/bdi.70080

**Published:** 2026-02-03

**Authors:** Juan Antonio García‐Carmona, Joshua Barnett, María Pilar Campos‐Navarro, Katie Mason, Jorge Simal‐Aguado, Sofia Pappa

**Affiliations:** ^1^ Department of Neurology Santa Lucia University Hospital Murcia Spain; ^2^ Group of Clinical and Experimental Pharmacology Institute for Biomedical Research of Murcia (IMIB) Murcia Spain; ^3^ Faculty of Health Sciences University of Murcia Murcia Spain; ^4^ West London National Health System (NHS) Trust London UK; ^5^ Department of Psychiatry Santa Lucía University Hospital Murcia Spain; ^6^ Department of Brain Sciences, Faculty of Medicine Imperial College of London London UK

**Keywords:** aripiprazole 1‐month, bipolar disorder, long‐acting injectable antipsychotics, paliperidone‐palmitate 1‐monthly, paliperidone‐palmitate 3‐monthly

## Abstract

**Background:**

This was a 4‐year mirror‐image study of adult patients diagnosed with bipolar disorder (BD) assessing the effects on treatment continuation and hospitalisation between aripiprazole 1‐month (A1M), risperidone‐LAI (R‐LAI) and the monthly and 3‐monthly formulations of paliperidone palmitate (PP1M, PP3M). We aimed to evaluate and compare the use of A1M, R‐LAI, and the monthly and 3‐monthly formulations of paliperidone palmitate (PP1M, PP3M) by using the change of number and length of hospitalisations 2 years before compared to 2 years after initiation of LAIs for continuers and discontinuers. Secondary outcomes were: (1) discontinuation rates at 2 years and reasons per LAI, (2) time to discontinuation per LAI, and (3) time to first hospitalisation per LAI.

**Results:**

A total of 122 BD were included; 74 continued LAI treatment at two years. Reasons for discontinuation were poor compliance (50%), ineffectiveness (43.2%), and tolerability issues (13.6%). Both time to individual LAI discontinuation and time to first hospital admission were significantly lower in the R‐LAI group. There was a significant overall reduction in the number and length of hospitalisations two years before and after LAI initiation, although multivariate logistic regression analysis showed that A1M, PP1M and R‐LAI were associated with an increased risk (OR = 1.89, 95% CI = 1.54–3.68, *p* = 0.015; OR = 1.63, 95% CI = 1.29–2.77, *p* = 0.022; OR = 3.08, 95% CI = 1.48–6.05, *p* = 0.008, respectively) of bed usage compared to PP3M. Last, study completers showed a considerable drop of 79% in number of hospital admissions and 83% in bed days (*p* = 0.001) as opposed to non‐completers.

**Conclusions:**

Study findings suggest that long‐acting antipsychotics such as A1M, PP1M, and particularly PP3M are associated with high retention and lower hospitalisation rates after 2 years of treatment in patients with BD.

## 1 Introduction

Bipolar disorder (BD) is a chronic clinical condition characterised by recurrent episodes of mania/hypomania and episodes of depressed mood. Its prevalence is around 1%–2% worldwide [1], and it is associated with a variety of other comorbid psychiatric and somatic conditions such as substance misuse, anxiety, personality disorders and/or cardiovascular disease [2]. BD patients suffer higher mortality rates, including increased risk of suicide [3]. The acute manic and depressive episodes considerably increase the mental health services resource utilisation and corresponding healthcare costs, with hospitalisations accounting for at least a quarter of the total direct costs of treating patients with BD [4]. Indeed, it has been reported that up to 75% of BD patients will suffer at least one relapse requiring hospitalisation in their lifetime [5].

Relapse prevention remains, therefore, a cornerstone in the management of BD. Both mood stabilisers and atypical antipsychotics are effective and widely used in the treatment of BD and, thus, included in most clinical guidelines either as monotherapy or in combination [6, 7, 8, 9]. Recent studies, however, revealed that between 10% and 60% of BD patients are non‐adherent to treatment, a main risk factor for relapse, rehospitalisation and suicide [10]. Long‐acting injectables (LAIs) antipsychotics have been shown to improve treatment adherence, patient experience and the collaborative process and reduce hospitalisations compared to oral antipsychotic medication in BD patients [11, 12, 13, 14]. However, they are usually prescribed as off‐label treatment for BD, given that only risperidone microspheres (R‐LAI) and aripiprazole once‐monthly (A1M) have been approved by the FDA for maintenance treatment in BD [15].

Hence, though LAIs are extensively used in routine clinical practice, studies evaluating their effectiveness in the management of BD are relatively limited and mostly confined to examining the outcomes of a single formulation or comparing two different formulations [16, 17, 18] while often including mixed cohorts of patients with BD, schizophrenia or schizoaffective disorder [17, 19, 20, 21, 22, 23]. A recent mirror‐image study, for example, of 36 patients with BD demonstrated beneficial effects of paliperidone palmitate 1‐month (PP1M) in reducing manic and mixed episodes [24] while a previous retrospective study showed that A1M may be more effective than haloperidol and risperidone‐LAIs in reducing hospital admissions [25]. Furthermore, a 2‐year mirror‐image study conducted in Turkey showed that A1M, PP1M and R‐LAI may reduce the number and length of hospital admissions compared to oral treatments, although it only included 17 BD patients treated with LAIs [26].

Nevertheless, there is a lack of large naturalistic studies comparing the effectiveness of the newest LAIs in real‐world clinical practice in patients with BD. Therefore, the primary aim of this study was to evaluate and compare the use of A1M, R‐LAI and the monthly and 3‐monthly formulations of paliperidone palmitate (PP1M, PP3M) by using the change in the number and length of hospitalisations 2 years before compared to 2 years after initiation of LAIs for continuers and discontinuers. Secondary outcomes were (1) discontinuation rates at 2 years and reasons per LAI, (2) time to discontinuation per LAI and (3) time to first hospitalisation per LAI.

## 2 Methods

We included patients with a diagnosis of BD initiated on a LAI formulation (index date) between 2015 and 2020. As previously described [27], patient data were collected from multiple sites, that is different psychiatric hospitals and mental health centres in Murcia (Spain) and London (UK). The study applied a mirror‐image design, whereby the comparator group is the same patient in a different time period, that is 2 years before and 2 years after a patient had been commenced with A1M, PP1M, PP3M or RLAI. Data collection was completed in 2022, allowing for a 2‐year post‐initiation follow‐up of all patients. The index date was defined as the earliest occurrence (first date) of a claim for one of the LAI therapies of interest during the study period.

In brief, eligibility criteria consisted of (a) adult patients (> 18), (b) with an established diagnosis of BD according to DSM‐V guidelines and (c) starting treatment with an LAI during the study period. To ensure a history of previous mental illness, the first diagnosis of BD had to be present for at least 2 years before the index date. Exclusion criteria were as follows: institutionalised patients, simultaneous use of two LAIs, a diagnosis of intellectual disability, schizophrenia, schizoaffective and autistic spectrum disorders, and patients with missing records covering the 4‐year study period. Patients were eligible if they had other comorbid mental health diagnoses such as personality disorder, anxiety or depression, and/or if they were taking other concomitant psychotropic medication.

### 2.1 Study Measures

Data collected at baseline (i.e., the index date) included demographic information such as age, sex, ethnicity, tobacco, illicit substance use and clinical data such as diagnosis, illness duration (years), the time to and reasons for LAI discontinuation, as well as the time to first psychiatric hospitalisation after the index date and the number and length of hospital admissions in the 2 years pre‐ and post‐initiation of the corresponding LAI. It was outside the scope of this study to collect data regarding disease severity, although the number of previous admissions was recorded as a proxy measure. Furthermore, we recorded and compared concomitant treatments, including the use of benzodiazepines, oral antipsychotics, antidepressants, mood stabilisers and anticholinergics, 2 years after the index date or the last treatment prior to LAI discontinuation. In order to compare the use of benzodiazepines and concomitant oral antipsychotics, we calculated the corresponding daily dose equivalents of diazepam or haloperidol (mg/day) as standards, as previously described [28].

### 2.2 Statistical Analysis and Confounding Factors

All analyses were performed using IBM SPSS Statistics version 21.0 (IBM Corp., Armonk, NY, USA). We expressed quantitative variables as means [standard error media (SEM)] and categorical variables as numbers (percentage). We assessed normality of distributions using histograms and the Shapiro‐Wilk test. Despite the distribution being normal, we used non‐parametric tests to avoid statistical biases. Therefore, sample basal characteristics were analysed by U‐Mann‐Whitney or Kruskal‐Wallis tests. Variables associated in the univariate analysis and variables with statistical trends (*p* < 0.1) were entered as factors with a multivariate logistic regression model to identify the risk factors or patients' characteristics associated with being administered concomitant benzodiazepines, antipsychotics or anticholinergics. Time to first hospital admission and LAI discontinuation after the index date were described using Kaplan‐Meier survival curves, and discontinuation reasons were summarised. Differences between LAIs in time to first hospital admission and discontinuation were examined using the Log‐rank test. Hazard ratios (HRs) and corresponding 95% confidence intervals (CIs) were also calculated using Cox proportional hazards models. In all tests, a two‐sided *p* < 0.05 was considered significant.

## 3 Results

A total of 122 BD patients with a mean age of 44.1 years using an LAI were included in the study. Sample characteristics are shown by LAI (A1M, *n* = 47; PP1M *n* = 34; PP3M *n* = 21; RLAI *n* = 20) in Table 1. No significant differences were observed between groups in age, marital status, substance use or illness duration. A statistical trend was noted for sex, with a higher proportion of women in the A1M group.

**TABLE 1:** Demographical, clinical data and concomitant treatments.

	Cohort *N* = 122	A1M *N* = 47	PP1M *N* = 34	PP3M *N* = 21	RLAI *N* = 20	*p*
Sex (%)						0.068
Women	49 (41)	27 (57)	8 (24)	8 (38)	6 (30)	
Men	73 (59)	20 (43)	26 (76)	13 (62)	14 (70)	
Age (y ± SEM)	44.1 ± 1.8	43.2 ± 2.1	42.2 ± 2.9	46.1 ± 3.6	48.4 ± 4.2	0.608
Disorder duration (y)	13.2 ± 1.0	12.6 ± 0.8	13.8 ± 1.1	13.3 ± 1.1	13.4 ± 1.6	0.937
Marital Status (%)						0.920
Single/Divorced	88 (72)	32 (68)	28 (82)	12 (57)	16 (80)	
Coupled/Married	34 (28)	15 (32)	6 (18)	9 (43)	4 (20)	
Tobacco & Drugs (%)						0.838
Tobacco	74 (61)	28 (57)	23 (67)	12 (60)	11 (55)	
Other Drugs*	48 (40)	21 (43)	12 (35)	7 (33)	8 (40)	
N benzodiazepines (%)	48 (39)					0.602
1	36 (30)	17 (35)	9 (26)	5 (24)	5 (25)	
2	11 (9)	6 (12)	1 (3)	1 (5)	3 (15)	
3	1 (1)	1 (2)	0	0	0	
N antipsychotics (%)	41 (34)					0.710
1	37 (30)	14 (29)	10 (29)	6 (29)	7 (35)	
2	4 (3)	1 (2)	1 (3)	0	2 (10)	
Anticholinergics (%)	11 (9)	2 (4)	3 (9)	1 (5)	5 (25)	0.042
Antidepressants (%)	7 (6)					0.855
1	6 (5)	2 (4)	1 (3)	1 (5)	2 (10)	
2	1 (1)	0	0	0	1 (5)	
Mood stabilisers (%)	62 (50)					0.999
1	54 (44)	19 (39)	15 (44)	12 (57)	8 (40)	
2	8 (6)	5 (10)	3 (9)	0	0	

* Including the use of alcohol, cannabis, cocaine or amphetamines without meeting criteria for substance use disorder.

### 3.1 Concomitant Treatments

LAIs were used as antipsychotic monotherapy in 66.4% of the cohort. The number of benzodiazepines, oral antipsychotics, anticholinergics, antidepressants and mood stabilisers used concomitantly are summarised by LAI in Table 1. In fact, 39% and 50% of the patients were simultaneously treated with benzodiazepines and mood stabilisers, respectively. The most prescribed benzodiazepine was lorazepam (30%), followed by clonazepam (21%), while the most prescribed mood stabiliser was lithium (61%), followed by valproate (32%). Overall, quetiapine was the most frequently prescribed oral antipsychotic (24%), followed by olanzapine (23%) and risperidone (23%). Only 9% of patients were treated with anticholinergics and 6% with antidepressants.

The risk factors associated with the concomitant use of benzodiazepines, oral antipsychotics and anticholinergics are outlined in Table 2. Multivariate logistic regression analysis revealed an increased risk of benzodiazepine usage (OR = 12.81, 95% CI = 6.25–16.35, *p* = 0.001) in the Spanish compared to the British cohort, while no differences were found in the use of oral antipsychotics and anticholinergics. Furthermore, PP1M and PP3M LAIs were associated with a decreased risk of benzodiazepine prescription (OR = 0.52, 95% CI = 0.36–0.72, *p* = 0.04; OR = 0.49, 95% CI = 0.44–0.74, *p* = 0.038, respectively) compared to A1M and R‐LAI. R‐LAI was also associated with an increased risk (OR = 2.67, 95% CI = 1.23–3.41, *p* = 0.039) of oral antipsychotic augmentation compared to A1M.

**TABLE 2:** Multivariate logistic regression analysis for concomitant treatments.

	OR	95% CI	*p*
BDZ intake			
Cohort			0.001
Spain	12.81	6.25–16.35	
UK	1		
Sex			0.450
Women	1		
Men	0.76	0.570–1.24	
LAIs			
A1M	1		
PP1M	0.52	0.36–0.72	0.040
PP3M	0.49	0.44–0.74	0.038
R‐LAI	1.17	0.89–1.34	0.761
Concomitant AP intake			
Cohort			
Spain	2.21	0.84–3.86	0.392
UK	1		
Sex			
Women	1		0.684
Men	1.55	0.63–2.13	
LAIs			
A1M	1		
PP1M	1.09	0.73–1.57	0.496
PP3M	0.92	0.62–1.20	0.302
R‐LAI	2.67	1.23–3.41	0.039
Anticholinergic intake			
Cohort			
Spain	1.19	0.91–1.43	0.734
UK	1		
Sex			
Women	1		0.570
Men	1.32	0.72–1.87	
LAIs			
A1M	1		
PP1M	1.39	0.80–1.65	0.457
PP3M	1.22	0.68–1.50	0.407
R.LAI	2.16	0.89–3.14	0.088

Abbreviations: A1M = aripiprazole 1‐month, AP = antipsychotic, BDZ = benzodiazepine, LAI = long‐acting‐injectable antipsychotic, PP1M = paliperidone palmitate 1‐month, PP3M = paliperidone palmitate 3‐month, R‐LAI = risperidone‐LAI, UK = United Kingdom.

**FIGURE 1:**
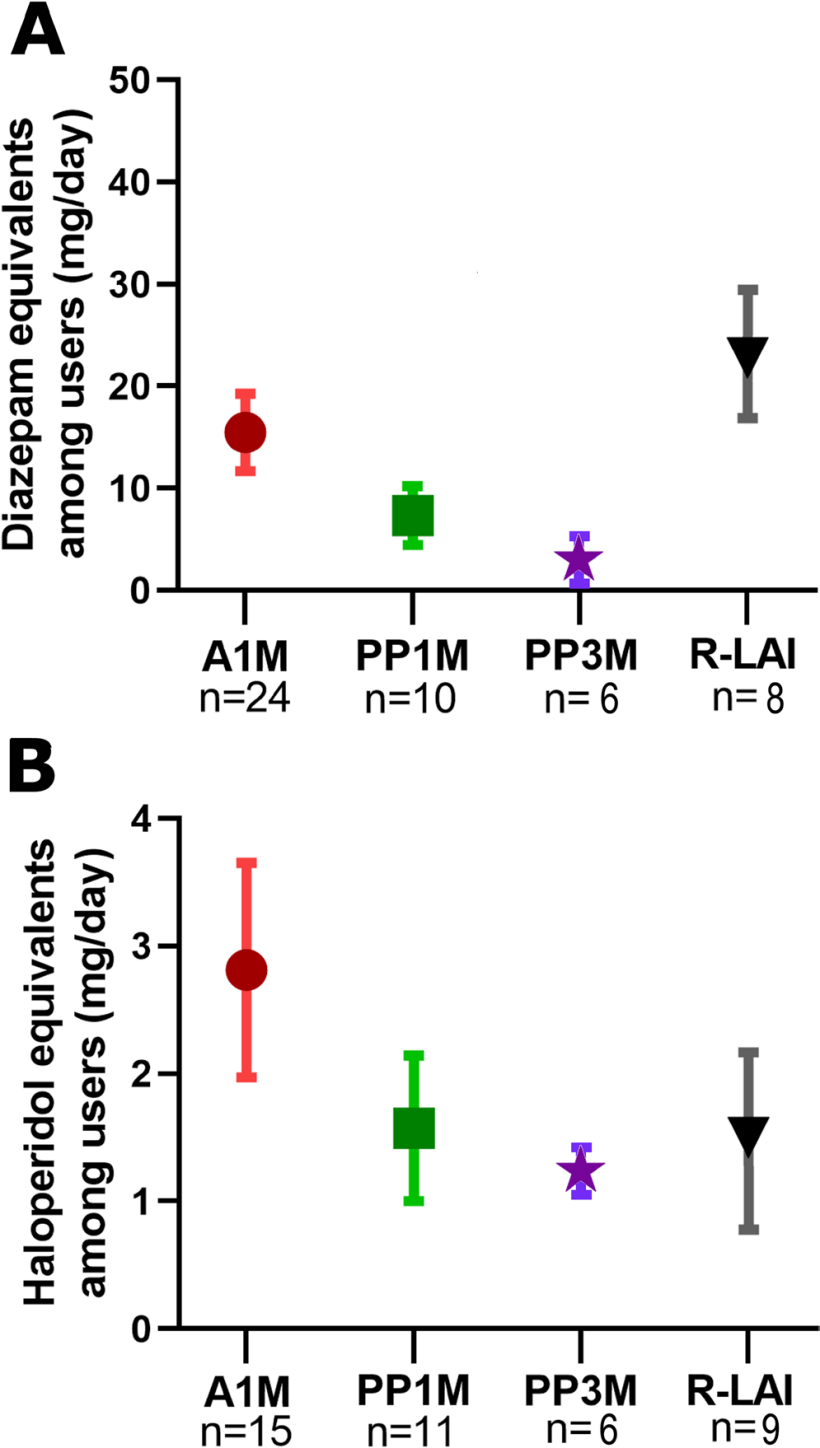
Psychiatric concomitant treatments. Doses of benzodiazepines and antipsychotics are showed as (A) diazepam and (B) haloperidol equivalents (mg/day) among users by LAI groups. Data are expressed as the mean SEM. A1M = aripiprazole‐1‐month; PP1M = paliperidone palmitate‐1‐month; PP3M = paliperidone palmitate‐3‐month; R‐LAI = risperidone‐LAI.

The mean daily doses of benzodiazepines and antipsychotics are shown in Figure 1 as (A) diazepam and (B) haloperidol equivalents. The mean daily dose of diazepam and haloperidol equivalents among users was 14.47 mg (±2.92) and 2.12 mg (±0.68), respectively. No statistical differences were found between LAI groups for either diazepam or haloperidol daily equivalents.

### 3.2 Time to and Reasons for LAI Discontinuation and Time to First Psychiatric Hospitalisation

In total, 48 out of 122 (39%) patients discontinued the LAI treatment at two years (A1M: *n* = 19 (39%); PP1M: *n* = 12 (35%); PP3M: *n* = 2 (10%); R‐LAI: *n* = 15 (75%)).

Indeed, the most frequent discontinuation reason was poor compliance [*n* = 22 (16%); A1M: *n* = 9 (18%); PP1M: *n* = 5 (15%); R‐LAI: *n* = 8 (40%)] followed by ineffectiveness [*n* = 19 (16%); A1M: *n* = 7 (20%); PP1M: *n* = 5 (15%); PP3M: *n* = 2 (10%); R‐LAI: *n* = 5 (25%)] and poor tolerance [*n* = 6 (5%); A1M: *n* = 2 (4%); PP1M: *n* = 2 (6%); R‐LAI: *n* = 2 (10%)]. Furthermore, one patient died in the A1M group due to a medical cause unrelated to the treatment.

**FIGURE 2:**
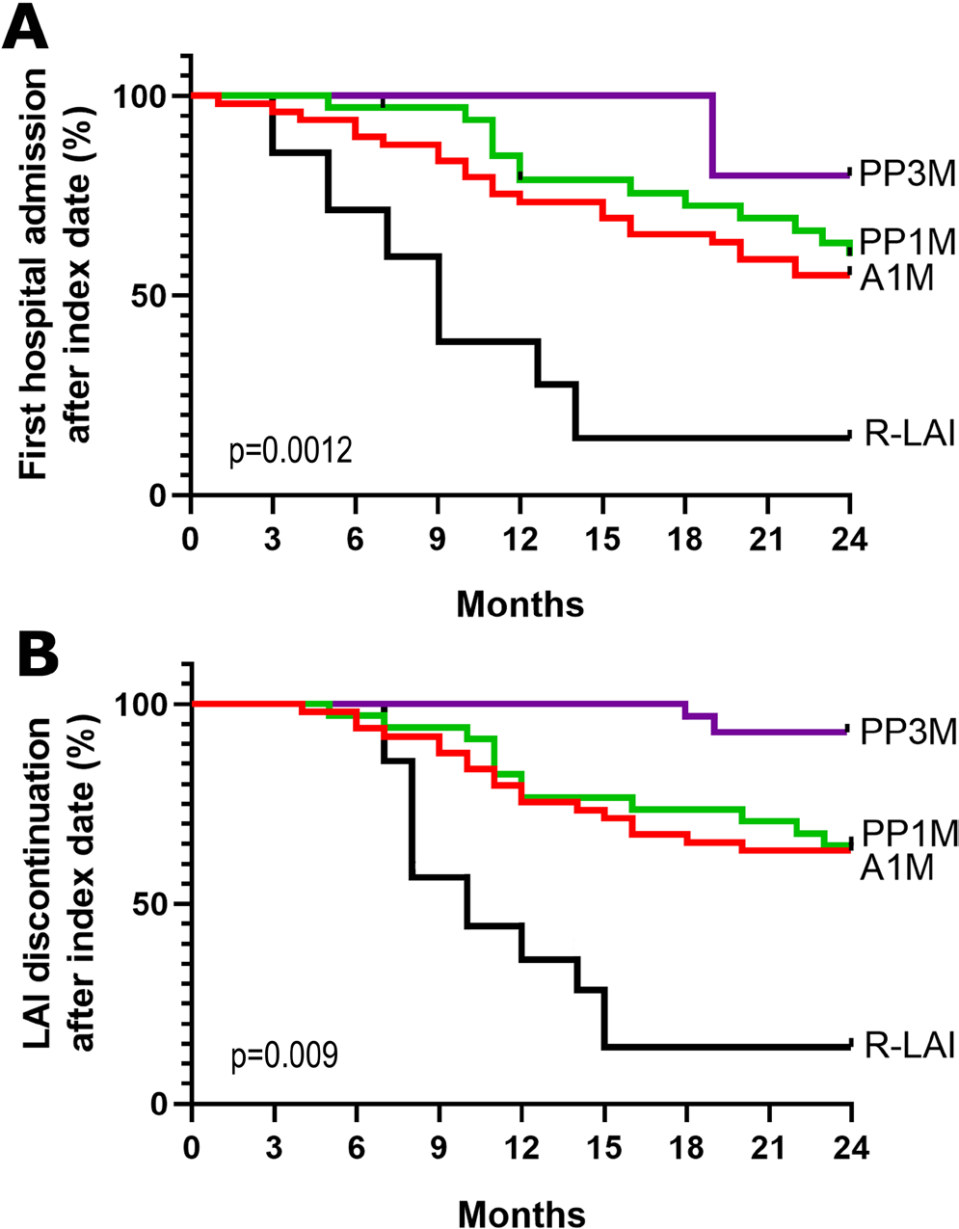
Kaplan‐Meier curves at 2 years after switching to LAI treatment. (A) Kaplan‐Meier curves comparing time until first hospital admission. (B) Kaplan‐Meier curves comparing time until LAI discontinuation. The *p* values were obtained from the log‐rank tests. A1M = aripiprazole‐1‐month; PP1M = paliperidone palmitate‐1‐month; PP3M = paliperidone palmitate‐3‐month; R‐LAI = risperidone‐LAI.

As shown in Figure 2A&B, Kaplan‐Meier curves revealed that both the time to treatment discontinuation and time to first hospital admission were significantly lower for the R‐LAI group compared to the other LAI groups (*p* = 0.009 and *p* = 0.0012, respectively).

### 3.3 Number of Hospital Admissions and Bed Days

**FIGURE 3:**
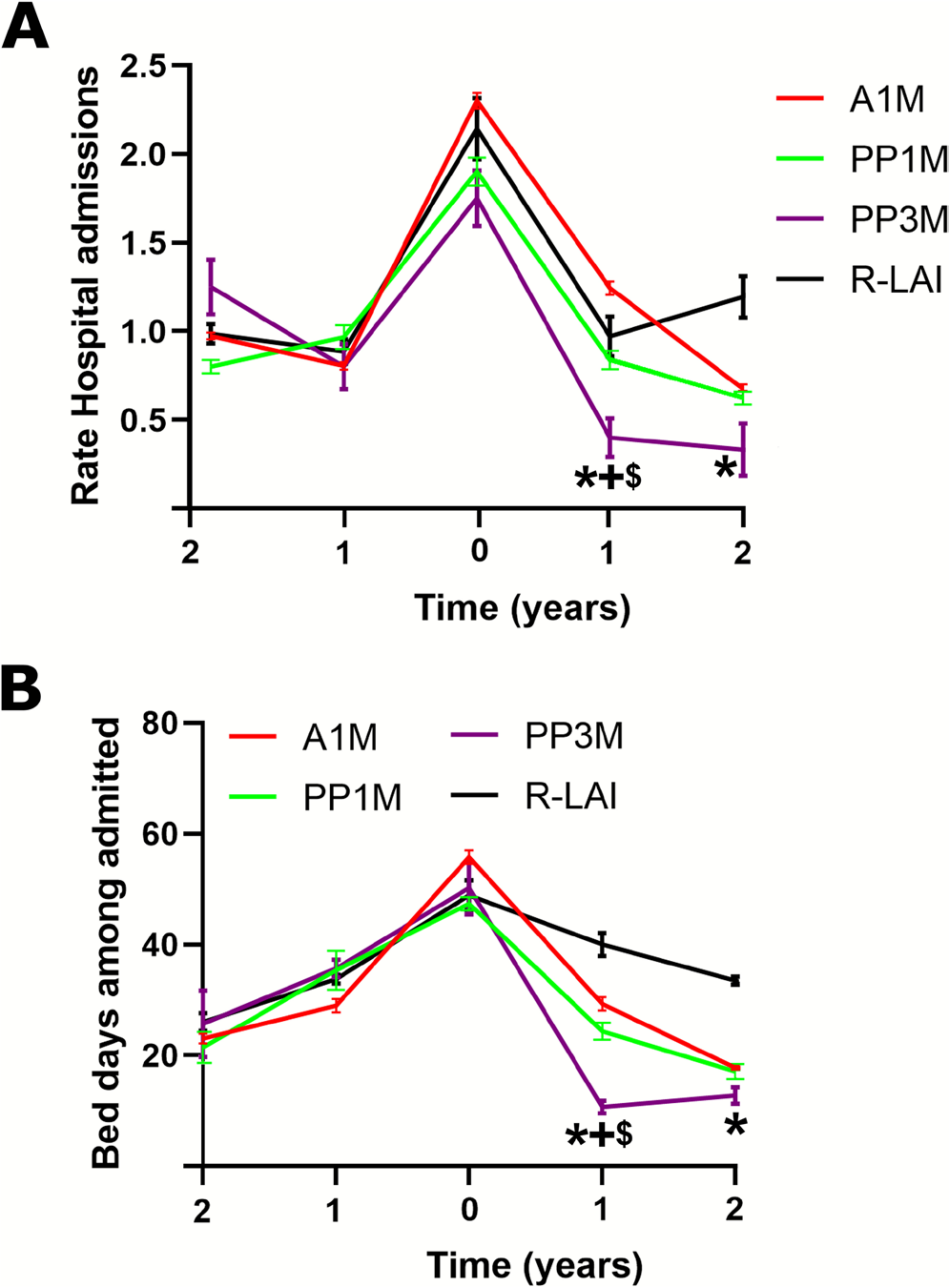
Hospital admissions and bed days among admitted. Mean of the ratio for patient and year about: (A) hospital admissions and (B) bed days by LAI groups. Data are expressed as the mean SEM. *p < 0.05 vs. R‐LAI group; + < 0.05 vs. A1M, $*p* < 0.05 vs. PP1M. A1M = aripiprazole‐1‐month; PP1M = paliperidone palmitate‐1‐month; PP3M = paliperidone palmitate‐3‐month; R‐LAI = risperidone‐LAI.

The number of hospital admissions (Figure 3A) was expressed as the mean number of hospitalisations per patient per year in each LAI group, and the length of stay (Figure 3B) was expressed as the mean number of bed days per year among admitted patients in each LAI group. Multivariate logistic regression analysis (Table 3) found significant LAI but no sex or cohort effects. In particular, it showed that A1M, PP1M and RLAI were associated with an increased risk (OR = 1.89, 95% CI = 1.54–3.68, *p* = 0.015; OR = 1.63, 95% CI = 1.29–2.77, *p* = 0.022; OR = 3.08, 95% CI = 1.48–6.05, *p* = 0.008, respectively) of being admitted to hospital compared with PP3M 2 years after the index date. The Bonferroni post hoc test revealed that PP3M was associated with significantly lower hospital admissions (0.39 ± 0.1) 1 year after the index date compared to A1M, PP1M (1.38 ± 0.04, *p* = 0.012; 0.79 ± 0.09, *p* = 0.05; respectively) and also compared to R‐LAI 1 and 2 years (1.05 ± 0.19, *p* = 0.05; 1.31 ± 0.21, *p* = 0.023; respectively) after initiating the LAI treatments.

**TABLE 3:** Multivariate logistic regression analysis for hospital admissions.

	OR	95% CI	*p*
Hospital admissions 1y after switching to LAI			
Cohort			
Spain	0.87	0.63–1.39	0.601
UK	1		
Sex			
Women	1		
Men	0.82	0.50–1.92	0.477
LAIs			
A1M	1.34	0.47–3.50	0.752
PP1M	0.74	0.42–2.41	0.487
PP3M	1		
R‐LAI	1.52	0.84–4.11	0.364
Hospital admissions 2y after switching to LAI			
Cohort			
Spain	1.07	0.91–1.23	0.495
UK	1		
Sex			
Women	1		
Men	0.84	0.67–1.27	0.591
LAIs			
A1M	1.89	1.54–3.68	0.015
PP1M	1.63	1.29–2.77	0.022
PP3M	1		
R‐LAI	3.08	1.48–6.05	0.008

Abbreviations: A1M = aripiprazole 1‐month, LAI = long acting‐injectable antipsychotic, PP1M = paliperidone palmitate 1‐month, PP3M = paliperidone palmitate 3‐month, R‐LAI = risperidone‐LAI, UK = United Kingdom.

**FIGURE 4:**
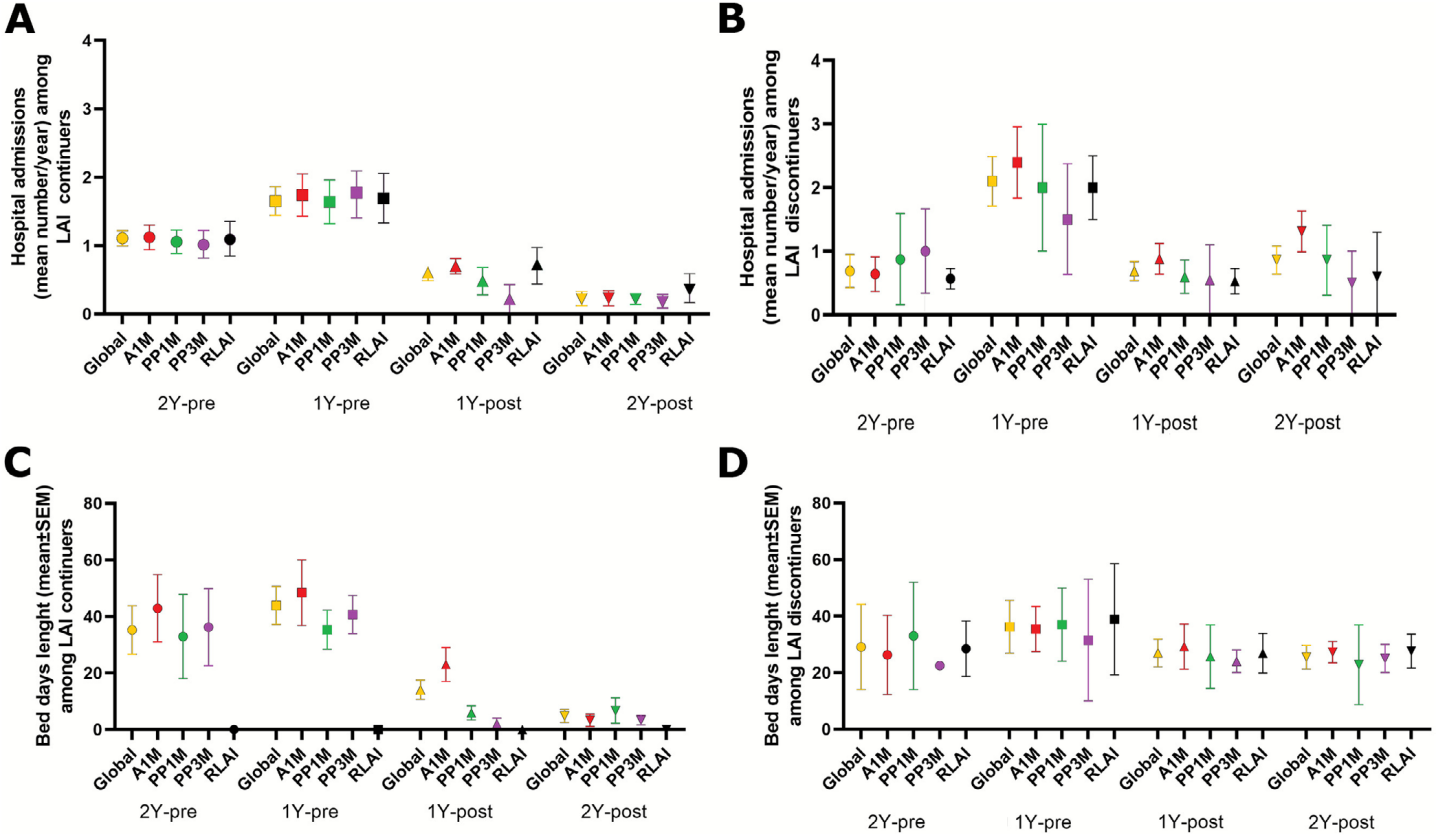
Hospital admissions and bed days among LAI continuers and discontinuers. Mean for LAI continued patients and year about: (A) hospital admissions and (C) bed days by LAI groups. Mean for LAI discontinue patients and year about: (B) hospital admissions and (D) bed days by LAI groups. Data are expressed as the mean. A1M = aripiprazole‐1‐month; PP1M = paliperidone palmitate‐1‐month; PP3M = paliperidone palmitate‐3‐month; R‐LAI = risperidone‐LAI.

For patients who continued with the LAI treatment for 2 years (*n* = 74) [A1M: *n* = 28 (57.1%); PP1M: *n* = 22 (64.7%); PP3M: *n* = 19 (90%); R‐LAI: *n* = 5 (25%)] the mean number of admissions decreased significantly from 1.19 ± 0.13 per patient in the 2 years prior to LAI initiation to 0.26 ± 0.12 in the 2 years after initiation (*p* = 0.018) (Figure 4A). Similarly, the mean length of admission (bed days) fell from 37.2 ± 8.6 days in the 2 years pre‐LAI initiation to 6.4 ± 1.7 days (*p* = 0.001) in the 2 years post‐LAI initiation (Figure 4C).

There was no significant improvement in hospitalisation rates (*p* = 0.710) and in the mean length of admissions (*p* = 0.83) in the 2 years after LAI initiation in the group of discontinuers (*n* = 48) compared to the 2 years pre‐LAI initiation, as shown in Figure 4B&D.

## 4 Discussion

This was a four‐year mirror‐image study that examined the risk of treatment discontinuation and psychiatric hospitalisation, as well as the use of concomitant medication among patients with BD who were initiated on different LAI treatments. Although naturalistic and mirror‐image studies demonstrate considerable strengths when assessing the impact and effectiveness of LAIs in patients with severe mental illness, to date, very few studies have been carried out evaluating the role and comparing the effects of the newest formulations, such as A1M, PP1M and PP3M in patients with BD.

### 4.1 Hospital Admissions and Bed Days

Our results demonstrated that the use of LAIs led to an overall significant reduction in both the number and length of hospital admissions at 2 years follow‐up. More specifically, BD patients treated with PP3M had a lower rate of hospital admissions compared with other LAIs, while R‐LAI performed the worst. When looking more specifically and comparing continuers (59%) at 2 years versus discontinuers (41%), the study completers showed a considerable drop of 81% in the number of hospital admissions and 86% in bed days (*p* = 0.001) as opposed to non‐completers who did not display statistically significant changes.

Previous findings deriving from large population‐wide cohorts in Finland demonstrated that LAIs and lithium are the most effective treatments in preventing relapse and hospital admissions in BD, with long‐acting antipsychotic formulations consistently outperforming their oral equivalents [13, 29]. Other naturalistic and mirror‐image studies showed similar results when comparing LAIs, such as A1M, R‐LAI and PP1M, and their corresponding oral formulations [18, 24, 30, 31, 32, 33]. In the few studies comparing different LAIs, risperidone LAI was found to decrease the rate of hospitalisation in BD patients compared to first‐generation antipsychotics LAIs in one study [16], while A1M was shown to decrease the rate of hospitalisation compared to both haloperidol and risperidone LAIs in a more recent study [25]. In contrast, this study found no differences between A1M and PP1M [25]. Other studies, including mixed cohorts of BD patients and patients with other mental health disorders, comparing different LAIs, showed comparable or even better effectiveness for the paliperidone formulations [27, 34, 35]. Furthermore, most of the aforementioned studies showed a reduction in the length of hospitalisations in BD patients treated with R‐LAI, A1M, and PP1M [18, 24, 26, 31, 32, 33] compared to the corresponding oral formulations. Only the study by Chan et al. failed to establish a significant difference in the number of days spent in hospital pre‐ and post‐initiation among 77 individuals with BD treated with LAIs [30].

As mentioned above, despite the extensive use in routine clinical practice, only R‐LAI and A1M LAIs have been approved by the FDA for the treatment of BD. Also, oral paliperidone is considered safe and effective as the first line in the treatment of manic episodes and second line in the maintenance treatment of bipolar disorder by the Canadian guidelines approved by the Canadian Network for Mood and Anxiety Treatments (CANMAT) [8]. Interestingly, a recent survey among psychiatrists in the United States showed that 69% of clinicians considered current guidelines to not adequately address all treatment options, in particular the role of LAIs in the treatment of BD patients [36]. In this regard, clinicians reported that they would consider initiating an LAI in BD patients when they have suffered a manic episode requiring hospitalisation and were previously treated with a number of antipsychotics. Furthermore, the following factors were considered as flags for non‐adherence in which initiating an LAI may be beneficial: family history of BD, anosognosia, concomitant substance use disorder, recent incarceration and polypharmacy [36].

### 4.2 Treatment Discontinuation and Causes

Overall treatment persistence with LAIs was favourable in our study, with 74 (60.6%) patients continuing at 2 years. Both the time to treatment discontinuation and the time to first hospital admission were significantly lower for the R‐LAI group compared to the other LAI groups. In addition, the longer‐ and shorter‐acting formulations of PP3M and R‐LAI displayed the better and worse retention rates at 90% and 25%, respectively, whereas PP1M and A1M were at 64.7% and 57.1%. Similarly, a recent study [37], comparing the efficacy and tolerability of PP1M vs. A1M in maintenance therapy of BD, showed that discontinuation rates at 1 year were overall higher for patients treated with A1M in comparison to PP1M (48% vs. 32.3%). In the same study, the main cause for LAI discontinuation in BD patients was tolerability issues, followed by ineffectiveness in the absence of significant group differences [37]. A previous study comparing R‐LAI with first‐generation LAIs found no differences in the discontinuation rate between groups [16]. A recent study showed a lower 1‐year discontinuation rate for PP1M compared to R‐LAI and olanzapine LAI in a cohort including BD patients, among others [18, 33]. This study reported the presence of side effects and patient refusal to continue with LAI treatment as the main reasons for discontinuation. Overall, the pattern and reasons for discontinuation of long‐acting antipsychotic treatments in patients with BD are not dissimilar to those observed in patients with schizophrenia [27, 38].

## 5 Limitations

This was an independently designed and conducted study that was not externally funded in order to minimise potential bias. Additional strengths include the 2‐year period of follow‐up and the involvement of multiple sites across two different European countries. Nonetheless, the findings of this study should be interpreted with caution due to its limitations. First, despite a relatively larger total sample size than most other mirror‐image studies in BD patients, the groups treated with PP3M and R‐LAI were small (21 and 20, respectively). Second, given that clinical severity was not recorded, it is likely that the results are neither generalisable nor causative. In fact, we inferred that the observed results were predominantly due to the use of LAIs, whereas we cannot exclude the potential effects of other concomitant medications, such as mood stabilisers, on treatment outcomes. Furthermore, this study did not include the recurrence/relapse events not requiring hospitalisation nor did it specify the nature or reason for hospitalisation, that is depressive, manic or mixed episode, with/without psychotic features. Third, the mirror‐image design has certain limitations in itself. For example, despite patients acting as their own control, disease duration, mood fluctuations and social/family circumstances can vary and impact treatment outcomes. Lastly, both A1M and R‐LAI are FDA approved for the maintenance treatment of BD patients. Therefore, it is possible that PP1M and PP3M are initiated off‐label in more severe patients.

## 6 Conclusions

Our results suggest that LAI formulations such as A1M, PP1M and particularly PP3M are effective treatment options in the management of bipolar disorder both in terms of treatment continuation as well as prevention of relapse and hospitalisations. Among the different options, R‐LAI performed the worst, possibly driven by the high discontinuation rates. Continued development of long‐ and longer‐acting antipsychotic treatments, their popularisation and inclusion into BD treatment guidelines alongside the clinical education of both patients and clinicians may help reduce hospitalisations rates and improve the management of bipolar patients. Future studies are needed to stratify those patients who are most likely to benefit from continuous treatment with long‐acting antipsychotics and to evaluate their effectiveness in larger sample sizes over extended observation periods beyond 2 years.

## Author Contributions

**Juan Antonio García‐Carmona** and **Sofia Pappa** wrote the initial draft of the manuscript; study design and interpretation of data; **Joshua Barnett**, **Katie Mason**, **María Pilar Campos‐Navarro** and **Jorge Simal‐Aguado** data collection and clinical consult. All authors wrote and reviewed the final version of the manuscript.

## Funding

The authors disclosed no receipt of financial support for the research, authorship and/or publication of this article.

## Ethics Statement

The study was approved by the Ethics Research Committee (ref. CE031914). Authorisation to access patients' medical records was granted by each site's Professional Services Directorate. No identifiable information was retained or is presented in this manuscript.

## Consent

The authors have nothing to report.

## Conflicts of Interest

Juan Antonio García‐Carmona received speaking honoraria from Janssen‐Cilag Inc. and Lundbeck, advisory honoraria from Teva and Angelini Pharma and research honoraria from Neuraxpharm and Roche, all of them outside the submitted work. S.P. has received honoraria as a consultant or speaker from Janssen‐Cilag Inc., Recordati, Sunovion, Rovi, Lundbeck and Otsuka and a research grant from Recordati, all outside the submitted work. Other authors declared no potential conflicts of interest with respect to the research, authorship and/or publication of this article.

## Data Availability

The datasets generated and analysed during the current study are available from the corresponding author upon reasonable request.
